# Comparison of Two Micro-osteoperforation Techniques on the Maxillary Anterior En Masse Retraction Rate in Adults: A Split-Mouth Quasi-experimental Study

**DOI:** 10.7759/cureus.86835

**Published:** 2025-06-27

**Authors:** Veerendra Kerudi, Shrutika Tamgadge, Utkarsha Raut, Amit Zope, Snehal Chintale, Sauravi Nimbalkar, Jay Patil, Seema Gupta

**Affiliations:** 1 Department of Orthodontics, Jawahar Medical Foundation's Annasaheb Chudaman Patil Memorial Dental College, Dhule, IND; 2 Department of Public Health Dentistry, Jawahar Medical Foundation's Annasaheb Chudaman Patil Memorial Dental College, Dhule, IND; 3 Department of Orthodontics, Kothiwal Dental College and Research Centre, Moradabad, IND

**Keywords:** acceleration, anterior, orthodontics, retraction, tooth movement

## Abstract

Introduction: Orthodontic treatment enhances both dental function and facial aesthetics; however, a longer treatment duration may lead to complications, prompting orthodontists to develop innovative methods to reduce treatment time without compromising the results. This study compared the rate of en masse retraction of maxillary anterior teeth using two different micro-osteoperforation (MOP) techniques: one with a bone drilling bur and a straight handpiece, and the other with a physiodispenser.

Materials and methods: A split-mouth quasi-experimental comparative study was conducted on 30 patients who visited the Department of Orthodontics and Dentofacial Orthopaedics between the ages of 18 and 30 years, requiring extraction of the maxillary first premolars. All the patients were treated with the 0.022 × 0.028-inch McLaughlin, Bennett, and Trevisi (MBT) prescription bracket kit (3M Unitek, Monrovia, CA, USA). After initial leveling and alignment, each patient was divided into two equal groups (n = 30) using a split-mouth design. On one side (Group A), MOP was performed using a bone-drilling bur with a straight handpiece, whereas on the other side (Group B), MOP was performed using bone-drilling burs with a physiodispenser. The anterior retraction rate was noted for six months. The Mann-Whitney U test was used for statistical analysis, with significance set at p < 0.05.

Results: The study demonstrated that the rate of en masse retraction was greater in the MOP with straight handpiece group than in the physiodispenser group at the end of the second month, third month, and fourth month; however, there was no nonsignificant difference between the two groups (p > 0.05) at the end of the first month and sixth month. At the end of the fifth month, the rate of en masse retraction was significantly greater in the MOP with a physiodispenser than in the straight handpiece group. The Mann-Whitney U test revealed no significant difference in total space closure between groups (Group A: 5.39 ± 0.73 mm; Group B: 5.17 ± 1.16 mm; p = 0.138). However, Group A demonstrated a significantly faster rate of space closure (0.90 ± 0.12 mm/month) compared to Group B (0.86 ± 0.19 mm/month; p = 0.043).

Conclusions: MOP with a straight handpiece significantly accelerated maxillary anterior en masse retraction compared with the physiodispenser.

## Introduction

Orthodontic treatment aims to enhance dental function and facial aesthetics by correcting malocclusions, often involving retraction of the anterior teeth following premolar extraction. While effective, conventional orthodontic treatment in adults typically lasts two to three years, longer than in adolescents, and is associated with increased risks of periodontal complications, enamel demineralization, root resorption, and reduced patient compliance [1,2]. The biological basis of orthodontic tooth movement relies heavily on periodontal ligament remodeling and alveolar bone turnover, processes regulated by inflammatory mediators and signaling pathways such as the receptor activator of nuclear factor kappa-B (RANK) and its ligand (RANKL) [3].

In response to the growing demand for shorter treatment durations without compromising clinical outcomes, several adjunctive techniques have been developed to accelerate tooth movement. These are broadly categorized into biological, physical, biomechanical, and surgical interventions [4]. Among these, minimally invasive surgical approaches such as micro-osteoperforation (MOP) have gained increasing attention. MOP involves creating small perforations in the alveolar bone to trigger local inflammation and stimulate bone remodeling, thereby accelerating tooth movement [5-7]. Both animal and human studies have reported increased rates of orthodontic tooth movement following MOP, associated with elevated levels of inflammatory cytokines, such as tumor necrosis factor-alpha (TNF-α) and interleukin-1 (IL-1), in the gingival crevicular fluid [6,8]. However, clinical evidence regarding its long-term efficacy and optimal technique remains limited and inconsistent.

MOP were initially popularized using specialized devices such as those developed by Propel Orthodontics, which are designed to deliver controlled and consistent perforations in the alveolar bone [7]. Although effective, these devices are expensive and may not be feasible for widespread clinical use, particularly in resource-limited settings. This has led to the exploration of more accessible alternatives to conventional dental equipment. Previous studies have evaluated MOP performed with bone-drilling burs using either a straight handpiece or a physiodispenser as viable, low-cost substitutes [9,10]. For example, Aboalnaga et al. [9] reported that an MOP with a straight handpiece and temporary anchorage devices (TADs) can effectively enhance tooth movement when performed correctly under controlled conditions. Similarly, Thomas et al. [10] demonstrated that using a physiodispenser with adequate torque control and irrigation results in favorable clinical outcomes with minimal patient discomfort. However, only a few studies have directly compared these two techniques.

Therefore, the present quasi-experimental split-mouth study was undertaken to compare the rate of maxillary anterior en masse retraction using two different MOP techniques: one performed with a bone-drilling bur and a straight handpiece, and the other with a bone-drilling bur and a physiodispenser. The objective of this study was to evaluate and compare the rate of tooth movement achieved with each technique over six months and determine which method facilitated faster retraction. The null hypothesis proposed that there would be no statistically significant difference in the rate of maxillary anterior en masse retraction between the two MOP techniques.

## Materials and methods

Study design and setting

A split-mouth quasi-experimental comparative study was conducted at the Department of Orthodontics and Dentofacial Orthopaedics, Jawahar Medical Foundation's Annasaheb Chudaman Patil Memorial Dental College, Dhule. Written informed consent was obtained from each patient before study initiation. The study spanned a period of 1.5 years, from November 2022 to June 2024. Ethical approval for this study was obtained from the Institutional Ethics Committee, with approval number EC/INST/2022/2959/O310.

This study was designed as a quasi-experimental split-mouth investigation rather than a randomized controlled trial (RCT). In contrast to RCTs, which require random allocation and the inclusion of a separate control group, this study involved all patients receiving both interventions, thereby eliminating the need for a non-intervention control. The allocation of techniques to each side was not randomized but was standardized or alternated to minimize selection bias while ensuring clinical feasibility. This within-subject comparison allowed for better control over inter-individual variability in the biological responses and treatment mechanics.

Sample size estimation

The sample size was determined based on data from a prior study by Raghav et al. [11], utilizing G Power software version 3.1.6 (Heinrich-Heine-Universität Düsseldorf, Düsseldorf, Germany). A minimum of 50 extraction sites was calculated, with a study power of 80%, an α error of 0.05, and a small effect size of 0.14, derived from an earlier study that accounted for repeated measures of maxillary canine movement following MOP. With an anticipated attrition rate of 10%, the final sample size was determined to be 60 extraction sites in 30 patients.

Eligibility criteria

Patients were selected based on the following inclusion criteria: male patients willing to undergo fixed appliance therapy, aged 18-30 years, Angle's Class I bimaxillary protrusion or Class II Division 1 malocclusion with no or minimal crowding of less than 2 mm requiring bilateral maxillary first premolar extractions, presence of all teeth except third molars, and healthy periodontal status (probing depth ≤ 3 mm, no active periodontal disease). Exclusion criteria included patients with a history of previous orthodontic treatment, gingival and periodontal diseases, systemic and medical illnesses, extreme skeletal Class II or Class III malocclusion, poor oral hygiene, craniofacial anomalies, morphological variation of any tooth in the maxillary arch, long-term use of medication interfering with orthodontic tooth movement such as phenytoin sodium, cyclosporine (antibiotics), anti-inflammatory drugs, systemic corticosteroids, calcium channel blockers, patients with habits of bruxism, parafunctional behaviors, and smoking.

Methodology

Each patient in this split-mouth comparative study was assigned to one of two groups. On one side (Group A, n = 30), MOP was performed using a bone-drilling bur with a straight handpiece, whereas on the other side (Group B, n = 30), MOP was performed using bone-drilling burs with a physiodispenser. Blinding of the patients and doctors administering the treatment was not possible at the time of intervention. Blinding was performed at the measurement and data analysis level.

All patients underwent thorough oral prophylaxis one week prior to their initial bonding and were instructed to chew on both sides of their teeth. All the patients were instructed to avoid chewing hard food after bonding. Each patient in the study was referred for extraction of the maxillary first premolars by the same surgeon to decrease variability. Extraction was performed six months prior to the MOP procedure to mitigate the effects of the regional acceleratory phenomenon (RAP), which may interfere with the MOP procedure [7].

Orthodontic treatment was initiated using a pre-adjusted edgewise appliance with the McLaughlin, Bennett, and Trevisi (MBT) bracket system (0.022-inch slot; 3M Unitek, Monrovia, CA, USA). Following the initial stages of leveling and alignment, self-drilling TADs (Unitek™ TAD, 1.8 × 8 mm) were positioned buccally between the upper second premolar and the first molar bilaterally for direct anchorage. Leveling and alignment were performed using the following sequence of wires: a 0.014-inch nickel-titanium (NiTi) wire (3M Unitek, Monrovia, CA, USA), followed by a 0.016 × 0.022-inch NiTi wire (3M Unitek, Monrovia, CA, USA). After leveling and alignment, a 0.019 × 0.025-inch stainless steel (SS) arch wire (3M Unitek, Monrovia, CA, USA) was placed for four weeks to allow the wire to become passive and remove the bias caused by the wire binding in the bracket slot.

**Figure 1:**
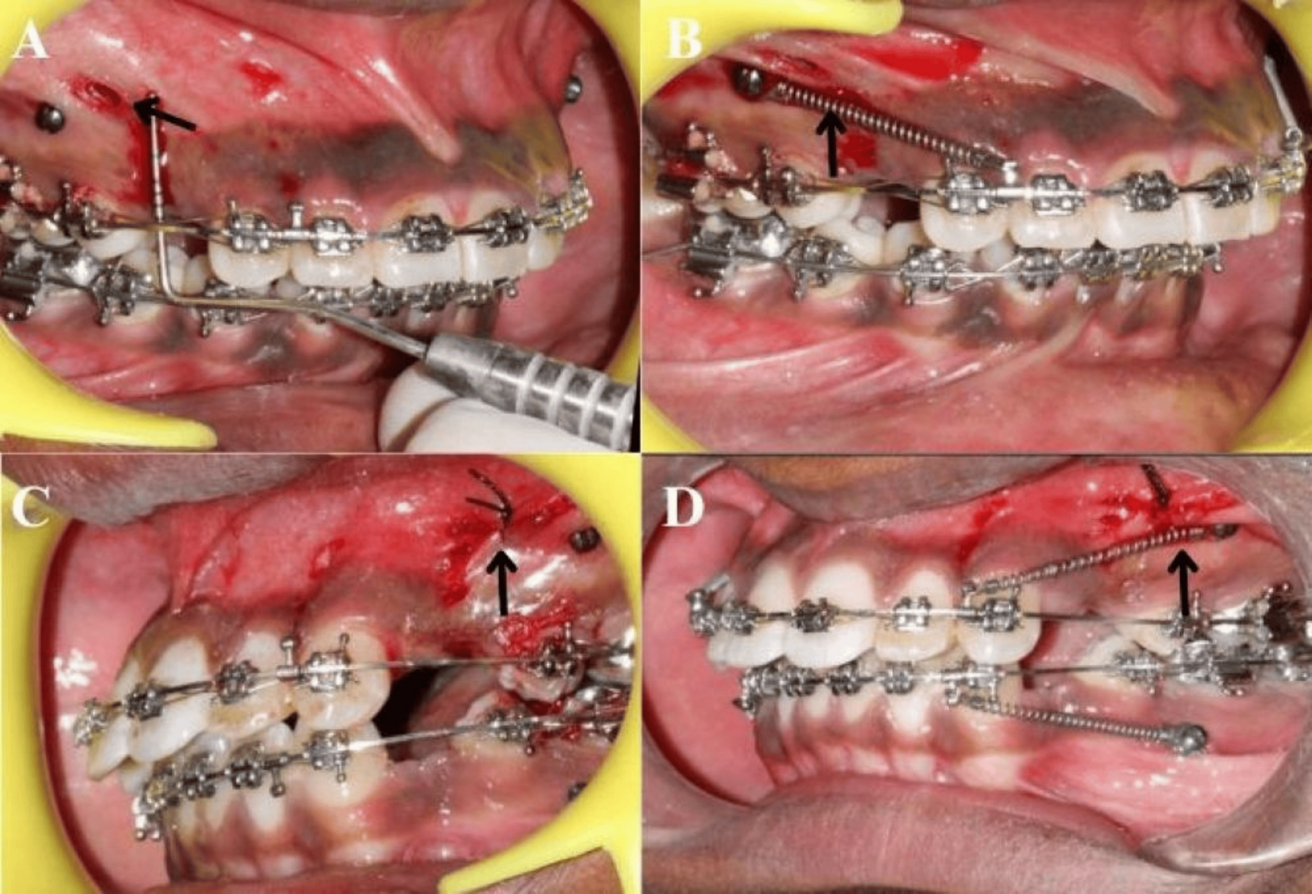
MOP and en masse retraction of maxillary anterior teeth in a split-mouth design. (A) MOP with a straight handpiece in Group A. (B) En masse retraction with NiTi closed-coil spring in Group A. (C) MOP with physiodispenser in Group B. (D) En masse retraction with NiTi closed-coil spring in Group B This figure represents intraoral images of a patient from our study, and is published with the patient's consent. MOP: micro-osteoperforation, NiTi: nickel-titanium

MOPs of size 1.5 mm (diameter) × 7 mm (length) were performed only once, just before the initiation of retraction. Three perforations were made mesial and distal to the canine and in the middle of the extraction space of the maxillary first premolar [9]. An L-shaped wire guide, with its upright portion measuring two-thirds of the length of the canine root [12], was affixed to the bracket through ligation. A permanent marker was used to segment the vertical wire guide into three equal parts, ensuring that each segment accommodated one MOP. For mesiodistal positioning, MOPs were executed at the midpoint of the extraction site (Figure 1).

MOP was performed to accelerate orthodontic tooth movement by creating small perforations in the alveolar bone at heights of 6 mm, 9 mm, and 12 mm from the gingival margin under copious saline irrigation to minimize heat and protect tissues. In Group A, MOP was conducted using a bone drilling bur (Edenta's Lindemann bur, Edenta AG, Switzerland) attached to an NSK S-Max M25 straight handpiece (NSK Nakanishi Inc., Japan) operating at 600 revolutions per minute (RPM) with a torque of 2.9 Ncm. In Group B, a physiodispenser (W&H Dentalwerk, Austria) was used at 500 RPM with a maximum torque of 50 Ncm paired with a similar bur. These tools ensure precise bone perforation with continuous saline cooling, preventing thermal damage and facilitating effective retraction.

Anterior en masse retraction in the maxillary arch was performed using NiTi closed-coil springs of 9 mm (G&H Orthodontics, Franklin, IN, USA), which were placed between the TADs and canine hook on both sides on the same day as the MOP was performed. A 150 g of force was applied using a Dontrix Gauge (Ortho Organizers, Carlsbad, CA, USA) on both sides with MOPs [13]. All patients were treated by a single experienced orthodontist with > 15 years of clinical experience to maintain treatment homogeneity. Bilateral first premolar extractions were performed atraumatically in the Department of Oral and Maxillofacial Surgery under local anesthesia (2% lidocaine with 1:100,000 epinephrine; Septodont, Saint-Maur-des-Fossés, France) by a trained, experienced single oral surgeon.

Outcomes assessment

Subsequent activations were organized once every month for six months to readjust the springs, assess the stability of the TADs, and identify any occlusal interferences that could occur during anterior retraction. The rate of maxillary anterior en masse retraction was assessed intraorally after performing MOP using two different methods by measuring the distance between the cusp tip of the canine and the second premolar with the help of a Digimatic Vernier Caliper (Mitutoyo Corp., Japan), which is known for its high accuracy (up to ± 0.03 mm). The unit of the retraction rate was mm.

Statistical analysis

Statistical analysis was done using a computer with the aid of SPSS Statistics version 25 (IBM Corp. Released 2017. IBM SPSS Statistics for Windows, Version 25.0. Armonk, NY: IBM Corp.). The normality of the data was checked using the Shapiro-Wilk test, and the data were found not to be normally distributed, justifying the use of nonparametric tests. Intergroup comparison of the en masse rate of retraction between the two dependent groups was performed using a Wilcoxon signed-rank test. Significance was assessed at a 5% level, meaning that a p-value of less than 0.05 was considered indicative of statistical significance.

## Results

**Figure 2:**
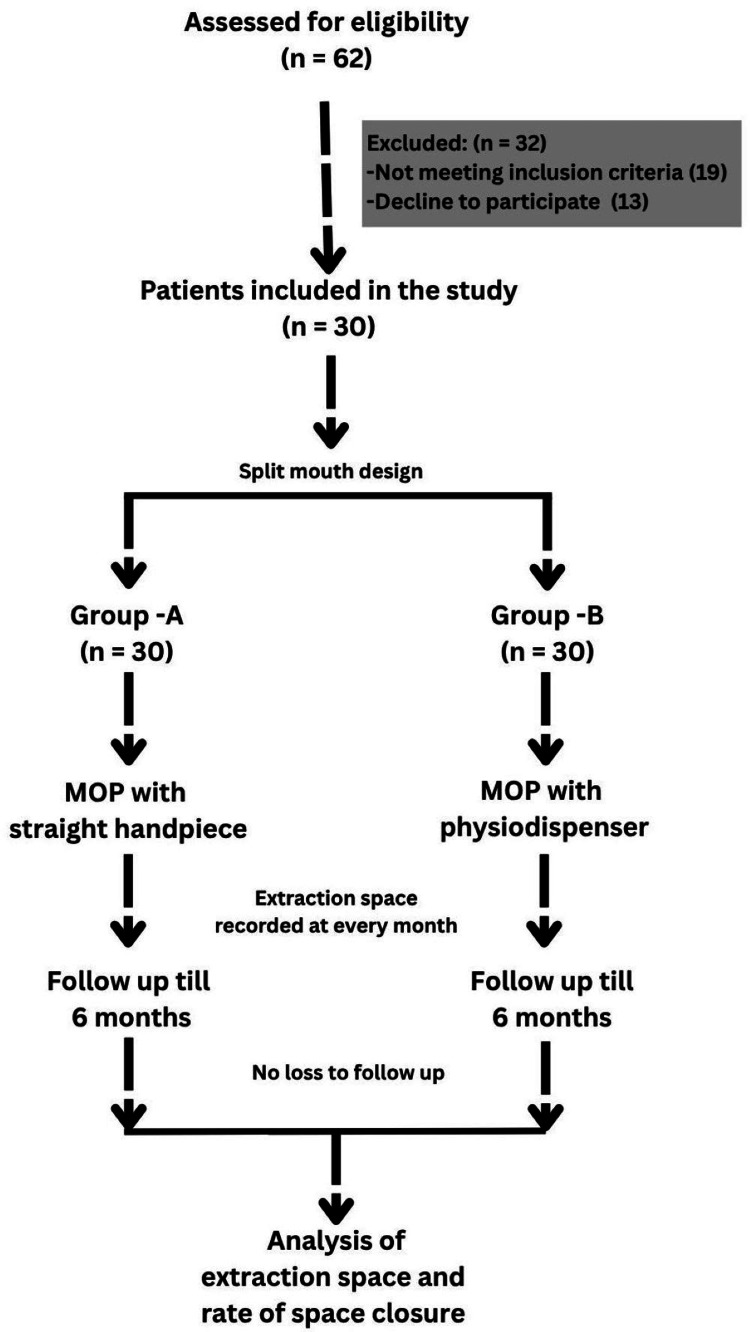
Study design MOP: micro-osteoperforation, Group A: MOP was performed using a bone-drilling bur with a straight handpiece, Group B: MOP was performed using bone-drilling burs with a physiodispenser

Figure 2 illustrates the study flowchart outlining the sequence of steps followed throughout the investigation. Of the 62 patients assessed for eligibility, 30 patients were evenly assigned to the two treatment groups in a split-mouth design. All the patients completed the study and were included in the final analysis. A total of 60 extraction sites were assessed in 30 patients to compare the rate of maxillary anterior en masse retraction between the two MOP techniques.

The sample consisted of 30 males with a mean age of 22.45 ± 3.12 years. The Wilcoxon signed-rank test revealed no significant baseline difference in the extraction space between groups (p = 0.081). At the end of the first month (M1), space closure remained comparable (p = 0.116). Significant differences emerged at the end of the second month (M2) (p = 0.001), at the end of the third month (M3) (p = 0.001), and at the end of the fourth month (M4) (p = 0.048). At the end of the fifth month (M5), Group B showed significantly greater closure (p = 0.001). By the end of the sixth month (M6), groups converged (p = 0.524). These findings suggested that Group A produced faster space closure, and therefore, the null hypothesis was rejected for this study (Table 1).

**Table 1:** Comparison of space closure (mm) between groups during en masse retraction of maxillary anterior teeth at different time points with the Wilcoxon signed-rank test *p-value < 0.05: significant, Group A: MOP was performed using a bone-drilling bur with a straight handpiece, Group B: MOP was performed using bone-drilling burs with a physiodispenser Data is presented in the form of mean ± SD MOP: micro-osteoperforation, SD: standard deviation

Timeline	Groups	Minimum	Maximum	Range	Mean ± SD	Z value	p-value
Baseline	Group A	10.60	15.97	5.37	11.89 ± 2.75	-2.46	0.081
	Group B	9.05	18.13	9.08	13.21 ± 3.01		
At end of first month (M1)	Group A	0.37	2.10	1.73	1.21 ± 0.63	1.57	0.116
	Group B	0.35	1.74	1.39	0.94 ± 0.46		
At end of second month (M2)	Group A	0.65	1.29	0.64	0.98 ± 0.19	-3.87	0.001*
	Group B	0.49	0.96	0.47	0.75 ± 0.18		
At end of third month (M3)	Group A	0.32	1.16	0.84	0.84 ± 0.26	-2.27	0.001*
	Group B	0.62	1.18	0.56	0.68 ± 0.15		
At end of fourth month (M4)	Group A	0.51	1.19	0.68	0.82 ± 0.24	-2.39	0.048*
	Group B	0.35	1.71	1.36	0.64 ± 0.43		
At end of fifth month (M5)	Group A	0.39	1.33	0.94	0.72 ± 0.27	-3.74	0.001*
	Group B	0.59	2.04	1.45	1.04 ± 0.44		
At end of sixth month (M6)	Group A	0.42	1.17	0.75	0.82 ± 0.21	-0.64	0.524
	Group B	0.49	1.26	0.77	0.80 ± 0.24		

The total space closure of extraction spaces represents the cumulative reduction of these spaces over a six-month period. The Wilcoxon signed-rank test revealed no significant difference in total space closure between groups (Group A: 5.39 ± 0.73 mm; Group B: 5.17 ± 1.16 mm; p = 0.138). However, Group A demonstrated a significantly faster rate of space closure (0.90 ± 0.12 mm/month) compared to Group B (0.86 ± 0.19 mm/month; p = 0.043). While both groups achieved similar total closure distances, Group A's narrower range (0.33 mm versus 0.67 mm) and higher mean rate suggested more consistent and efficient tooth movement (Table 2).

**Table 2:** Comparison of space closure between groups during en masse retraction of maxillary anterior teeth with the Wilcoxon signed-rank test *p-value < 0.05: significant, Group A: MOP was performed using a bone-drilling bur with a straight handpiece, Group B: MOP was performed using bone-drilling burs with a physiodispenser Data is presented in the form of mean ± SD MOP: micro-osteoperforation, SD: standard deviation

Timeline	Groups	Minimum	Maximum	Range	Mean ± SD	Z value	p-value
Total space closure (mm)	Group A	4.19	6.15	1.96	5.39 ± 0.73	-1.48	0.138
	Group B	3.41	7.43	4.02	5.17 ± 1.16		
Rate of space closure (mm/month)	Group A	0.70	1.03	0.33	0.90± 0.12	-1.69	0.043*
	Group B	0.57	1.24	0.67	0.86 ± 0.19		

## Discussion

An extended treatment duration can discourage patients from undergoing orthodontic treatment [1]. Prolonged orthodontic treatment increases the risk of complications, including dental caries, periodontal problems, white spot lesions, and root resorption, due to the continuous application of orthodontic forces [2]. Various pharmacological and surgical strategies have been investigated to accelerate tooth movement [4]. While pharmacological agents such as prostaglandins and vitamin D promote bone remodeling, their clinical application is limited by systemic side effects, short half-lives, uneven distribution, and inconsistent evidence regarding their efficacy [14]. Surgical approaches to accelerate tooth movement include both invasive and minimally invasive procedures. Although these methods can be effective with relatively fewer side effects, they tend to be more costly and invasive and may cause discomfort for patients [15].

In this study, a minimally invasive approach was selected using MOP. The MOP procedure showed increased localized bone turnover. Due to perforations, there is continuous circulation of inflammatory chemokines and cytokines at the localized application site, significantly increasing the number of osteoclasts, which in turn leads to bone resorption. Additionally, enhanced bone remodeling extends beyond the moving tooth, affecting the tissues surrounding adjacent teeth [7]. The procedure is cost-effective, patient-friendly, and practical for clinical application. Although these methods may reduce treatment duration, further research is needed to enhance their safety, effectiveness, and long-term outcomes [7].

A study conducted by Erdenebat et al. [8] on rats demonstrated that the use of MOPs accelerated tooth movement and was associated with rapid cementum regeneration and formation of periodontal ligament tissue. Ruan et al. [1] found that age affects the rate of tooth movement, with adults exhibiting a reduced bone remodeling capacity compared to children, who have a higher RANKL ratio. To mitigate the effects of age and sex on tooth movement rates, male patients aged 18-30 years were selected for this study. A split-mouth design was chosen to minimize the biological variability among patients. The bias in variations in bone density between the maxilla and mandible was eliminated in this study by evaluating only the maxillary arch.

Based on a prior systematic review conducted by Al-Khalifa et al. [7], it has been indicated that occlusal forces can considerably influence the velocity of dental movement. To mitigate this issue, we carefully selected patients exhibiting similar malocclusion types and instructed them to engage in bilateral mastication. Individuals exhibiting bruxism were excluded from this study. Three maxillary MOPs significantly increased pro-inflammatory cytokines and accelerated tooth movement by 2.3 times [7]. Therefore, in the present study, it was decided to incorporate three MOPs: mesial, distal to the canine, and midway in the extraction space.

The results of the present study indicated that a statistically significantly greater en masse retraction of the anterior teeth was noticed with a straight handpiece, which could be due to a more pronounced or optimally timed RAP compared to the physiodispenser, potentially due to differences in mechanical parameters such as rotational speed and torque. The straight handpiece, operating at 600 RPM with a torque of 2.9 Ncm, likely produces a different pattern of bone micro-trauma than the physiodispenser, which operates at 500 RPM with a higher torque of 50 Ncm. The lower torque and slightly higher RPM in the straight handpiece may create more superficial and uniform perforations, potentially optimizing the inflammatory response required for RAP. In contrast, a physiodispenser’s higher torque could result in deeper or more aggressive perforations, possibly leading to a prolonged healing phase that delays peak bone remodeling. This hypothesis is supported by the non-significant differences observed after the second month, suggesting that the initial advantage of the straight handpiece diminishes as the RAP effect stabilizes over time [16].

The results of this study partially align with those of previous studies on MOPs. For example, Alikhani et al. [17] reported a 2.3-fold increase in the rate of canine retraction with MOPs compared with controls, although their study did not compare different MOP techniques. However, the lack of sustained significant differences beyond the second month in our study contrasts with some reports suggesting prolonged effects of MOPs [18]. This discrepancy may be attributed to differences in MOP protocols, such as the number, size, or depth of perforations, or variations in patient populations.

The comparison between the straight handpiece and physiodispenser is novel, as few studies have directly evaluated the different methods of MOP delivery. Raghav et al. [11] used a bone-drilling bur but did not compare delivery systems, focusing instead on MOP versus non-MOP controls. They concluded that MOP resulted in faster tooth movement only during the first month of treatment. This could be due to the transient nature of RAP phenomena [19]. The current study’s focus on the two mechanical delivery methods provides a unique contribution to the literature, highlighting the potential impact of device-specific parameters on treatment outcomes.

Clinical implications

The finding that the straight handpiece group exhibited a significantly higher rate of retraction suggests that this technique may offer a clinical advantage in scenarios where early acceleration of tooth movement is desired. For instance, in patients with bimaxillary protrusion or Class II Division 1 malocclusion that requires rapid anterior retraction, a straight handpiece may be preferred to expedite the treatment. However, the choice between the two methods might depend on factors such as equipment availability, operator experience, and patient comfort.

Limitations of the study

Despite its strengths, this study had some limitations that warrant consideration. A sample size of 30 patients, while adequately powered based on prior data, might limit the generalizability of the findings. Additionally, the study was limited to male patients aged 18-30 years with specific malocclusions, which may not accurately reflect the broader orthodontic population. Future studies should include diverse demographics, such as female patients and a wider age range, to assess the generalizability of the findings. The study did not evaluate pain perception associated with the MOP procedure or inflammatory markers and was performed only on the maxillary arch. Future research could explore whether multiple MOP applications over the treatment period can sustain the initial acceleration observed with a straight handpiece. Additionally, histological or imaging studies could elucidate the biological mechanisms underlying the differences in retraction rates, particularly the role of torque and RPM in modulating the RAP.

The exclusion of patients with systemic conditions or those using medication was necessary to control for confounders, but it limited the applicability of the findings to medically complex patients. Future studies should investigate the safety and efficacy of MOPs in such populations to provide ethical considerations. The cost-effectiveness and patient comfort associated with each technique were not evaluated in this study; however, these factors are critical for informed clinical decision-making. Finally, the non-inclusion of a control group was a significant drawback of this study.

## Conclusions

This split-mouth quasi-experimental study demonstrated that MOP, using a bone-drilling bur with a straight handpiece, resulted in a significantly higher rate of maxillary anterior en masse retraction compared to a physiodispenser. These findings suggest that the straight handpiece may offer a clinical advantage in accelerating tooth movement. The choice between these methods should consider clinical practicality, operator expertise, and patient preference. Further research is needed to investigate the long-term effects and broader applicability of this approach.
